# Association between dietary approaches to stop hypertension (DASH) diet and hyperuricemia among Chinese adults: findings from a nationwide representative study

**DOI:** 10.1186/s12937-023-00845-w

**Published:** 2023-03-29

**Authors:** Qianrang Zhu, Lianlong Yu, Yuqian Li, Qingqing Man, Shanshan Jia, Beibei Liu, Wenqi Zong, Yonglin Zhou, Hui Zuo, Jian Zhang

**Affiliations:** 1 National Institute for Nutrition and Health, Chinese Center for Disease Control and Prevention, Beijing, China; 2 NHC Key Laboratory of Trace Element Nutrition, National Institute for Nutrition and Health, Chinese Center for Disease Control and Prevention, Beijing, 100050 China; 3 Shandong Center for Disease Control and Prevention, Jinan, China; 4 Jiangsu Provincial Center for Disease Control and Prevention, Nanjing, China; 5 School of Public Health, Medical College of Soochow University, Suzhou, China

**Keywords:** Dietary approaches to stop hypertension, Serum uric acid, Hyperuricemia, China adult chronic disease and nutrition surveillance

## Abstract

**Background:**

Certain foods and food groups could positively or negatively impact serum uric acid (SUA) levels. However, evidence on the holistic dietary strategy to prevent and control hyperuricemia (HUA) development remains limited.

**Objective:**

The aim of this research work was to explore the association of dietary approaches to stop hypertension (DASH) diet with SUA levels and odds of HUA among Chinese adults.

**Methods:**

This research premise included 66,427 Chinese adults aged 18 and above who were part of the China Adult Chronic Disease and Nutrition Surveillance in 2015. Dietary consumptions were assessed via the household condiment weighing approach and a three-day, 24-hour dietary recall. Total fat, saturated fat, calcium, protein, potassium, cholesterol, magnesium, fiber, and sodium were all adopted to calculate the DASH score (score range, 0–9). The associations of DASH score with SUA levels and odds of HUA were evaluated using multiple linear and logistic regression models, respectively.

**Results:**

We established that a higher DASH score was linked with a lower SUA levels (β = − 0.11; 95% CI: − 0.12, − 0.1; *p* < 0.001) and odds of HUA (OR = 0.85; 95% CI: 0.83, 0.87; *p* < 0.001) after adjustment for age, sex, ethnicity, education status, marital status, health behaviours and health factors. The association of the DASH diet with odds of HUA was stronger among men (p-interaction = 0.009), non-Han Chinese (p-interaction< 0.001) as well as rural residents (p-interaction< 0.001).

**Conclusions:**

Our results illustrate that the DASH diet was remarkably negatively with SUA levels and odds of HUA in the Chinese adult population.

**Supplementary Information:**

The online version contains supplementary material available at 10.1186/s12937-023-00845-w.

## Introduction

Hyperuricemia (HUA) is a metabolic disease caused by the metabolism disorder and/or impaired renal uric acid excretion [1]. HUA is the main pathogenic factor for gout [1], and might participate in the onset and progress of hypertension, type 2 diabetes, chronic kidney disease, metabolic syndrome, as well as cardiovascular disease [2–5]. Over the last few decades, the occurrence of HUA has risen in developed along with developing countries [6]. In China, HUA has been increasing in prevalence from 8.4 to 14% in adults during 2009 to 2019, and has become a public health problem to be addressed [7].

Diet, as an important determinant of serum uric acid (SUA) levels [8], could have a positive or negative impact on the prevention of HUA [9]. Intake of alcohol, sugary sweetened beverages (SSB) and some purine-rich foods for instance red meat and shellfish triggers the increase of SUA levels [10]. In contrast, consumption of some specific elements of diet including low fat milk [10], vitamin C [11] and dietary fiber [12] may reduce SUA levels. However, focusing on single nutrient or food may not comprehensively reflect the impact of overall dietary quality on health outcomes due to the diversity and complexity of food intake [13]. Previous studies indicated that the conventional low-purine (i.e., low-protein) dietary approach to lowing SUA levels may worsen insulin resistance and elevate levels of fasting blood glucose, triglycerides and LDL cholesterol as a result of compensatory higher intake of refined carbohydrates or fats [14, 15]. Therefore, a holistic dietary strategy is needed to better prevent, as well as control the development of HUA.

The Dietary Approaches to Stop Hypertension (DASH) diet, which underscores foods rich in protein, calcium, fiber, potassium along with magnesium, and consisting of fruits, low-fat dairy products, vegetables, nuts, beans, as well as whole grains, whereas limiting foods harboring high saturated fat and sugar, was originally developed for hypertension management [16]. The DASH diet has also been found to reduce the odds of some HUA related comorbidities for instance cardiovascular disease, chronic kidney disease, metabolic syndrome, as well as type 2 diabetes [17]. HUA is causally related with insulin resistance [18], and thus might also be improved by DASH diet, which has the potential to enhance insulin sensitivity [19]. Two intervention studies conducted in the U.S. have observed significant reductions in SUA levels among participants with hypertension or gout following DASH diet [20, 21]. However, in a randomised controlled trial (RCT), the benefit effects of DASH diet on SUA levels was only found among African Americans hypertensive patients who had a a higher baseline SUA levels [22]. So far, the existing epidemiological research evidence on this issue in the general population is very limited. A previous cross-sectional study conducted in Tangshan city, China observed that adherence to DASH diet has been documented to be linked with a lower odds of HUA among Chinese general adult population [23]. But, in this study, all participants were residents of the same community, who are mainly Northern Han Chinese. Thus, the focus of our research was to explore link between a DASH diet with SUA levels and the odds of HUA in 66,427 Chinese adults using the most recent nationally representative data.

## Materials and methods

### Study population

Data were derived from the China Adult Chronic Disease and Nutrition Surveillance (CACDNS) 2015. The CNCDNS is a national representative cross-sectional research work which was designed to assess food intake, health status and lifestyle behaviors of Chinese adults aged 18 and above. Participants were enrolled from 31 provinces in China (except Hong Kong, Macao, and Taiwan) via a multistage stratified cluster random sampling approach along with a probability proportionate to population size sampling technique, which has been documented previously in details [24]. A total of 69,909 individuals with available data of dietary intake, lifestyle behaviors, medical history, laboratory results along with physical examination, were included in this study. Participants with implausible total energy intakes (< 800 kcal/day (kcal/day) or > 4500 kcal/day)(*n* = 2169), pregnant women (*n* = 141), cancer patients(*n* = 1019), as well as nursing mothers(*n* = 153) were further excluded from further analysis. The excluded participants did not show significant differences regarding socio-demographics, BMI, or serum uric acid levels. Finally, 66,427 participants were involved in the analysis.

At the start of the survey, all participants granted a written informed consent form. The Chinese Center for Disease Control and Prevention’s Ethical Committee authorized the research work protocols (No. 201519-B).

### Assessment of dietary scores

The 3-day (two weekdays coupled with one weekend day) food record approach was used to determine dietary intake of each individual. The amounts of edible oil and each condiments (such as salt, sugar and other ingredients) used at the household were assessed by the three-day household condiments weighing method, using a uniformly calibrated electronic dietary scale with a precision of 2 g [25]. The interviewers consisted of public health physicians drawn from local community health centers as well as disease control and prevention centers who had been trained on dietary data recording. In the course of the household interviews, staff utilized standard forms of dietary recalls harboring picture aids, as well as food models. Throughout the survey, participants were required not to modify their eating or lifestyle habits. Dietary data along with the Chinese Food Composition Table were utilized to estimate nutrient and energy intake [26, 27].

The DASH diet score for each participant were calculated based on the Mellen et al. formula in this study [28]. Briefly, nine targeted nutrients including total fat, fiber, saturated fat, calcium, protein, potassium, cholesterol, magnesium, and sodium were identified for DASH goals. Micronutrient goals were expressed per 1000 kcal. We assigned a value of 1 if the participant met the DASH target of a nutrient, a value of 0.5 reflected meeting a nutrient’s intermediate target, whilst 0 reflected no target met. By adding all nutrient targets met, the total DASH score is then calculated, which varies from 0 to 9. The detailed target for each nutrient is illustrated in Supplementary Table 1.

### Ascertainment of outcome

This study focused on serum urate concentration as the primary outcome. SUA contents of ≥7.0 mg/dL in men or ≥ 6.0 mg/dL in women defined HUA [29]. We collected blood samples from consenting participants following an overnight fasting of 10–12 hours, and spun and fractionated into plasma, as well as serum within 0.5–1.0 hours post collection. Serum specimens were stored at − 80 °C for downstream analyses. SUA contents were further measured with the Hitachi Automatic Platform 7600 (Hitachi Co., Tokyo, Japan). Each of the measures listed above was performed in a laboratory by specialists under stringent quality control.

### Measurement of other covariates

Information was obtained from each participant on demographic characteristics (date of birth, region, gender, education along with marital status), health behaviors (smoking status, level of physical activity, and drinking status) coupled with medical histories with the use of a questionnaire completed by an interviewer.

In previous studies, these factors, in addition to dietary factors, have been found to be associated with the odds of hyperuricemia in Chinese adults [7]. Attainment of education was stratified into three classes; less than high school, high school and higher education. Marital status consisted of either living single (widowed, separated, or divorced) and not single (living with partner or married). Physical activity was summarized and expressed in metabolic equivalents (METs) minutes/week, and then classified into three categories: vigorous (MET> 3000), moderate (600 ≤ MET≤3000), or sedentary (MET< 600) [30]. Status of smoking along with drinking were classified as yes (current or former) or no (never).

Body height and weight were assessed via calibrated electronic digital scales with a precision of 0.1 cm and 0.01 kg, respectively. Body mass index (BMI) was then computed as kg/m^2^. Systolic and diastolic blood pressures were assessed for three times with a 1-minute interval between each measurement with an automatic measurement device (Omron HBP-1300; OMRON Healthcare, the Netherlands). In the analysis, the average of three measurements was utilized.

The contents of total cholesterol, fasting blood glucose, high-density lipoprotein cholesterol, low-density lipoprotein cholesterol, as well as low-density lipoprotein cholesterol were assessed on the Hitachi Automatic Platform 7600 (Hitachi Co., Tokyo, Japan). Diabetes was defined by having a self-reported history of diabetes mellitus or having a fasting glucose level of more than 7.0 mmol/L. The criteria of defining hypertension consisted of a self-reported hypertension history, or an average SBP or an average SBP ≥ 140 mmHg or DBP ≥90 mmHg. Dyslipidemia was defined as total cholesterol (TC) level ≥ 5.7 mmol/L, or high-density lipoprotein cholesterol (HDL-C) level < 1.04 mmol/L, or low-density lipoprotein cholesterol (LDL-C) level ≥ 3.64 mmol/L, or current use of antihyperlipidemic drugs. Participants with any of the above diseases were defined as having a chronic disease noncommunicable diseases (NCDs). The medication use was included in the medical history referred to whether the participants were taking anti-hypertensive drugs, anti-hyperglycemic drugs, and lipid-lowering drugs. Urologic disease, for instance kidney stones, chronic nephritis prostatitis, was assessed via self-report.

### Statistical analysis

Participants were stratified into quartiles on the basis of the DASH score beginning with the lowest (the 1st quartile) to the highest (the 4th quartile). Basic features and dietary intake of the study participants were reported with the use of median (interquartile range [IQR]) or means (standard deviation [SD]) for continuous variables and number (percentage) for categorical variables. As the proportion of participants without dietary intake of dairy products (85.9%), sugar-sweetened beverages (SSB) (96.4%) is relatively large, we used number (percentage) instead of median (interquartile range [IQR]) to express the intake status of these food types. Differences between groups were analyzed via Kruskal-Wallis test for continuous variables and chi-square test for categorical variables. We further used generalized linear regression and logistic regression models to explore the the association of DASH diet scores with SUA levels and odds of HUA. Multivariable regression models were adjusted for these covariates: Model 1: adjusted for age, sex; Model 2: further adjusted for body mass index, region (city, rural), ethic (han, others), marital status (married or living with partner, living alone), education (less than high school, high school, higher than higer school), physical activity (sedentary, moderate, vigorous), smoking status (yes, no) and alcohol consumption (never, former, current), NCDs status (yes, no), medication use (yes, no), urologic disease status (yes, no). *P* values for trend (P-trend) were computed via the median within each group to assess the ordered relation across groups of the DASH score for both the continuous (SUA) and binary (HUA) outcomes. We also tested interaction between the DASH diet and age (< 45y, > = 45 y), sex, ethic group (Han, others), region (urban, rural), body mass index (< 24, > = 24) and health status (with NCDs, without NCDs) in the fully adjusted model. Statistically significance was defined as *P* < 0.05. The statistical analyses were conducted using the R software version 3.6.3.

## Results

This study enrolled 66,427 adults, including 31,920 males and 34,507 females. The overall prevalence of HUA in the entire sample was 14% (18% in men; 10.3% in women). Table 1 shows the characteristics of the study participants across the DASH score quartiles. Participants with higher DASH scores were more likely female, greater than 60 years old, rural residents, non-Han Chinese, with less than a high school education, not single, vigorous physical activity, not smoke or drink alcohol, with NCDs and use concomitant medications, without the urological disease. Relative to participants in the lowest quartile, those with DASH score in the highest quartile had a lower level of BMI and SUA levels.

**Table 1:** Sample characteristics of participants according to quartile of the DASH score (*n* = 66,427)

Characteristics	DASH score				*P*-value
	Q1(0–1)	Q2(1.01–1.99)	Q3(2–2.5)	Q4 (2.51–9)	
n	19,548	10,506	21,974	14,399	
Male (%)	9807 (50.2%)	5128 (48.8%)	10,441 (47.5%)	6674 (46.4%)	< 0.001
Age (years)	52 (42, 62)	52 (43, 63)	54 (44, 64)	56 (46, 65)	< 0.001
Age group					< 0.001
< 45	6549 (33.5%)	3130 (29.8%)	5808 (26.4%)	3517 (24.4%)	
45–59	7377 (37.7%)	4007 (38.1%)	8361 (38%)	5216 (36.2%)	
≥60	5622 (28.8%)	3369 (32.1%)	7805 (35.5%)	5666 (39.3%)	
Region					< 0.001
City	8745 (44.7%)	4571 (43.5%)	8632 (39.3%)	5057 (35.1%)	
Rural	10,803 (55.3%)	5935 (56.5%)	13,342 (60.7%)	9342 (64.9%)	
Ethic group					< 0.001
Han	17,447 (89.3%)	9478 (90.2%)	19,678 (89.6%)	12,516 (86.9%)	
Others	2101 (10.7%)	1028 (9.8%)	2296 (10.4%)	1883 (13.1%)	
Education					< 0.001
<High School	14,916 (76.3%)	8231 (78.3%)	17,860 (81.3%)	11,905 (82.7%)	
High School	2899 (14.8%)	1401 (13.3%)	2692 (12.3%)	1674 (11.6%)	
>High School	1733 (8.9%)	874 (8.3%)	1422 (6.5%)	820 (5.7%)	
Marital status					< 0.001
Not single	17,918 (91.7%)	9690 (92.2%)	20,227 (92.3%)	13,274 (92.2%)	
Single	1630 (8.3%)	816 (7.8%)	1697 (7.7%)	1125 (7.8%)	
Physical Activity Level					< 0.001
Sedentary	3272 (16.7%)	1711 (16.3%)	3772 (17.2%)	2465 (17.1%)	
Moderate	6341 (32.4%)	3374 (32.1%)	6820 (31%)	4315 (30%)	
Vigorous	9935 (50.8%)	5421 (51.6%)	11,382 (51.8%)	7619 (52.9%)	
Smoking					< 0.001
Never	12,592 (64.4%)	6876 (65.4%)	14,594 (66.4%)	9929 (69%)	
Former	1224 (6.3%)	758 (7.2%)	1575 (7.2%)	1072 (7.4%)	
Current	5732 (29.3%)	2872 (27.3%)	5805 (26.4%)	3398 (23.6%)	
Drinking (yes)					< 0.001
Never	11,777 (60.2%)	6381 (60.7%)	13,986 (63.6%)	9959 (69.2%)	
Former	1799 (9.2%)	997 (9.5%)	2006 (9.1%)	1177 (8.2%)	
Current	5972 (30.6%)	3128 (29.8%)	5982 (27.2%)	3263 (22.7%)	
NCDs (yes)					
Total	10,130 (51.8%)	5513 (52.5%)	12,188 (55.5%)	8055 (55.9%)	< 0.001
Diabetes (yes)	1673 (8.6%)	924 (8.8%)	1968 (9%)	1269 (8.8%)	0.558
Hypertension (yes)	7602 (38.9%)	4268 (40.6%)	9737 (44.3%)	6608 (45.9%)	< 0.001
Dyslipidemia (yes)	4538 (23.2%)	2307 (22%)	4824 (22%)	2991 (20.8%)	< 0.001
Urologic disease (yes)	2041 (10.4%)	1115 (10.6%)	2320 (10.6%)	1387 (9.6%)	0.019
Hyperuricemia (yes)	3687 (18.9%)	1812 (17.2%)	3214 (14.6%)	1906 (13.2%)	< 0.001
Medication history					
Total	2859 (14.6%)	1699 (16.2%)	3736 (17%)	2671 (18.5%)	< 0.001
Hypoglycemic (yes)	682 (3.5%)	376 (3.6%)	794 (3.6%)	537 (3.7%)	0.702
Antihypertensive (yes)	2281 (11.7%)	1351 (12.9%)	3018 (13.7%)	2165 (15%)	< 0.001
Lipid-lowering (yes)	441 (2.3%)	293 (2.8%)	681 (3.1%)	501 (3.5%)	< 0.001
BMI (kg/m2)	23.8 (21.5, 26.3)	23.9 (21.6, 26.5)	24.1 (21.7, 26.5)	24 (21.7, 26.6)	< 0.001
SUA (mg/dL)	5.2 (4.3, 6.3)	5.1 (4.2, 6.2)	5 (4.1, 6)	4.8 (4, 5.8)	< 0.001

Continuous variables were reported as median (IQR). Categorical variables were reported as N (%)

*Abbreviations*: *DASH* Dietary Approaches to Stop Hypertension, *BMI* Body mass index, *SUA* Serum uric acid, *NCDs* noncommunicable diseases, *IQR* interquartile range

*P*-value were calculated by Kruskal-Wallis tests for continuous variables, andχ^2^ test for categorical variables

The dietary intake information including nutrients and food categories of the study participants according to DASH score quartiles is presented in Table 2. Participants with DASH score in the highest quartile showed a dietary trend consisting of a higher intake of protein, fiber, protein, fiber, magnesium accompanied by a higher ingestion of vegetables, nuts and legumes and whole grains. In contrast, those participants with lowest DASH score showed a higher consumption of saturated fat, total fat, cholesterol, sodium accompanied by a higher ingestion of red along with processed meat and fruit. The fraction of participants with dietary intake of dairy products was lower among participants with DASH score in the highest quartile whereas the reverse was true with sugar-sweetened beverages (SSB).

**Table 2:** Dietary intake of the participants across quartile of the DASH score (*n* = 66,427)

Characteristics	DASH score				*P*-value
	Q1(0–1)	Q2(1.01–1.99)	Q3(2–2.5)	Q4 (2.51–9)	
n	19,548	10,506	21,974	14,399	
Energy (kcal/day)	1823.5 (575)	1800.8 (583.89)	1750.9 (580.5)	1731.4 (586.3)	< 0.001
Daily nutrients intake /1000 kcal					
Saturated fat (%energy)	7 (5)	5 (3.5)	3 (2.7)	2 (2.1)	< 0.001
Total fat (%energy)	45 (9.1)	40 (9.7)	36 (12)	24 (9)	< 0.001
Protein (%energy)	12 (2.5)	12 (3.1)	12 (3.7)	13 (3.8)	< 0.001
Cholesterol (mg)	189.3 (100)	147.9 (104.4)	115.6 (118.6)	89.7 (112.3)	< 0.001
Fiber (g)	4.4 (1.8)	5.1 (2.3)	5.5 (2.8)	7.3 (4.6)	< 0.001
Magnesium (mg)	119.9 (24.8)	132.9 (32.2)	140.4 (40.6)	174.1 (90.1)	< 0.001
Calcium (mg)	174.9 (68.9)	185.5 (78.9)	184.8 (90.9)	211.4 (124.6)	< 0.001
Potassium (mg)	729.3 (195.5)	760.6 (228.8)	756.7 (257.9)	882.9 (341.5)	< 0.001
Sodium (mg)	3143.1 (2058.1)	3106.6 (2106.5)	3227.3 (2573)	2692.7 (2234.7)	< 0.001
Daily food intake /1000 kcal					
Vegetables (g)	138 (72.3)	139.8 (79.1)	137.3 (88)	153.9 (108.3)	< 0.001
Fruit (g)	18.2 (34.8)	20.9 (39)	19.5 (39.6)	21 (45.4)	< 0.001
Dairy (yes)	2835 (14.5%)	1614 (15.4%)	2982 (13.6%)	1948 (13.5%)	< 0.001
Nuts and legumes (g)	9.5 (12.6)	12 (15.2)	12.3 (17.1)	15 (23)	< 0.001
Whole grains (g)	113.2 (39.8)	127.8 (47)	144.4 (53.4)	178.3 (59.6)	< 0.001
Red and processed meat (g)	52.2 (32.2)	39.3 (29.3)	27.2 (29.2)	21 (28)	< 0.001
SSB (yes)	768 (3.9%)	400 (3.8%)	710 (3.2%)	504 (3.5%)	0.01

Continuous variables were reported as means (SD). Categorical variables were reported as N (%)

*Abbreviations*: *DASH* Dietary Approaches to Stop Hypertension, *SSB* sugar-sweetened beverages

*P*-value were calculated by Kruskal-Wallis tests for continuous variables, andχ^2^ test for categorical variables

Table 3 provides the multiple linear regression data, exhibiting the association of DASH score with alteration in SUA contents. Following correcting for prospective various confounders (Model 2), an inverse association of the DASH score with SUA contents was reported (β = − 0.11; 95% CI − 0.12, − 0.1). In contrast to the first quartile, the β along with the 95% CI of Q2, Q3, and Q4 were − 0.08 (− 0.1, − 0.05), − 0.23 (− 0.25, − 0.2), and − 0.34 (− 0.37, − 0.31) (P-trend < 0.001) in Model 2.

**Table 3:** Multivariable associations between DASH score and serum uric acid concentrations among Chinese adults in 2015 (*n* = 66,427)

DASH score	Model 1	Model 2
	β (95% CI)	β (95% CI)
Continuous exposures	-0.12 (−0.13, −0.11)	−0.11 (−0.12, −0.1)
Q1	Ref	Ref
Q2	−0.07 (−0.1, −0.04)	−0.08 (−0.1, −0.05)
Q3	−0.22 (−0.25, −0.2)	−0.23 (−0.25, −0.2)
Q4	−0.35 (−0.38, −0.32)	−0.34 (−0.37, −0.31)
P-trend	< 0.001	< 0.001

Model 1: adjusted for age, sex

Model 2: further adjusted for BMI, region (city, rural), ethic (han, others), marital status (married or living with partner, living alone), education (less than high school, high school, higher than higer school), physical activity (sedentary, moderate, vigorous), smoking behavior (never, former, current), alcohol consumption (never, former, current), NCDs status (yes, no), medication use (yes, no), urologic disease status (yes, no)

*Abbreviations*: *DASH* Dietary Approaches to Stop Hypertension, *BMI* body mass index, *NCDs* noncommunicable diseases

Table 4 provides the multivariable-adjusted ORs along with the 95% CI for HUA on the basis of the DASH score. Following correcting for prospective confounders (Model 2), more adherence to the DASH dietary pattern exhibited beneficial effects on HUA (OR = 0.85; 95% CI 0.83, 0.87). Relative to the lowest quartile of DASH score, the ORs along with 95% CI for HUA were 0.88 (0.83, 0.94), 0.72 (0.68, 0.76) and 0.65 (0.61, 0.69) (P-trend < 0.001) in Model 2.

**Table 4:** Multivariable associations between DASH score and odds of hyperuricemia among Chinese adults in 2015 (*n* = 66,427)

DASH score	Model 1	Model 2
	OR (95% CI)	OR (95% CI)
Continuous exposures	0.85 (0.84, 0.87)	0.85 (0.83, 0.87)
Q1	Ref	Ref
Q2	0.9 (0.84, 0.96)	0.88 (0.83, 0.94)
Q3	0.74 (0.7, 0.78)	0.72 (0.68, 0.76)
Q4	0.66 (0.62, 0.7)	0.65 (0.61, 0.69)
P-trend	< 0.001	< 0.001

Model 1: adjusted for age, sex

Model 2: further adjusted for BMI, region (city, rural), ethic (han, others), marital status (married or living with partner, living alone), education (less than high school, high school, higher than higer school), physical activity (sedentary, moderate, vigorous), smoking behavior (never, former, current), alcohol consumption (never, former, current), NCDs status (yes, no), medication use (yes, no), urologic disease status (yes, no)

*Abbreviations*: *DASH* Dietary Approaches to Stop Hypertension, *BMI* body mass index, *NCDs* noncommunicable diseases

Table 5 provides the data of stratified assessment for the relationship of DASH score with HUA risk. The association between DASH score and odds of HUA were similar in sub-groups stratified by age group (p-interaction = 0.723), body mass index (p-interaction = 0.372) and health status (p-interaction = 0.762), and was stronger among male (p-interaction = 0.009; OR = 1.05; 95% CI = 1.01–1.1), non-Han Chinese (p-interaction < 0.001; OR = 0.83; 95% CI = 0.76–0.91) as well as rural residents(p-interaction < 0.001; OR = 0.89; 95% CI = 0.84–0.95).

**Table 5:** Stratified analysis of the associations between the DASH score and odds of hyperuricemia among Chinese adults in 2015

		Case/N	OR (95%Cl)	p-interaction
Age group				0.723
	< 45y	2946/19004	0.88 (0.84, 0.91)	
	> = 45 y	7673 /47423	0.85 (0.82, 0.88)	
Gender				0.009
	Male	6624 (32073)	0.85 (0.83, 0.88)	
	Female	3995 (34354)	0.87 (0.85, 0.9)	
Ethic group				< 0.001
	Han	9362 (59119)	0.87 (0.85, 0.89)	
	Others	1257 (7308)	0.74 (0.7, 0.79)	
Region				< 0.001
	Urban	4852 (27005)	0.88 (0.86, 0.9)	
	Rural	5767 (39422)	0.76 (0.72, 0.81)	
BMI				0.372
am	< 24	3776 (33581)	0.87 (0.84, 0.89)	
	> = 24	6843 (32846)	0.86 (0.84, 0.88)	
Health status				0.762
	With NCDs	7702 (37159)	0.86 (0.83, 0.88)	
	Without NCDs	2917 (29268)	0.86 (0.84, 0.88)	

Model: adjusted for age, sex, BMI, region (city, rural), ethic (han, others), marital status (married or living with partner, living alone), education (less than high school, high school, higher than higer school), physical activity (sedentary, moderate, vigorous), smoking behavior (never, former, current), alcohol consumption (never, former, current), NCDs status (yes, no), medication use (yes, no), urologic disease status (yes, no)

*Abbreviations*: *DASH* Dietary Approaches to Stop Hypertension, *BMI* body mass index, *NCDs* noncommunicable diseases

## Discussion

In this nationally representative cross-sectional research work, we established that the DASH diet was inversely linked to SUA contents and corresponding odds of HUA among 66,427 Chinese adults. These associations remained following adjusting for prospective confounders consisting of age, gender, body mass index, region, race, marital status, education background, physical activity, smoking status, drinking status, NCDs status and urological disease status.

As far as we know, this is the first research premise to reveal the beneficial impacts of DASH diet on SUA levels and odds of HUA among Chinese adults using a nationally representative sample. Our results were consistent with findings from several intervention studies conducted in the United States. In a 30-day randomized, crossover feeding trial, the DASH diet reduced SUA levels in adults with prehypertension or stage I hypertension, and the effect was stronger among those who had a baseline SUA level ≥ 7 mg/dL with an average decrease of 1.29 mg/dL in SUA levels which almost achieve the effect of pharmacologic urate-lowering therapy [20]. In another randomized, controlled, cross-over pilot investigation, the dietitian-directed, DASH-patterned groceries was found to reduce SUA levels modestly among gout participants not on urate-lowering drugs in 4 weeks [21]. Results from a parallel arm, controlled-randomized trial demonstrated that the DASH diet lowered SUA in the course of 30 days in prehypertensive or hypertensive adults, with a sustained effect at 90 days [31]. In addition, by partially replacing typical diet with DASH foods, a randomized trial lowered SUA contents in hypertensive African American with higher baseline contents of serum UA [22]. A similar observation was reported by two observational studies (refs). The first was a prospective cohort investigation of with 26 years of follow-up established that higher adherence to DASH diet in American males result in lower gout risk, illustrating that its influence of decreasing uric acid contents in participants with HUA corresponds a lower risk of gout [32]. The second was a cross-sectional investigation conducted in residents of the Kailuan community of Tangshan City, China, who are mainly Northern Han Chinese, found the DASH diet was linked to a low likelihood of developing HUA in adults [23]. Our study reconfirmed this beneficial effect in a sample of Chinese adults from different ethnic groups and regions throughout China, and thus could be generalizable to the entire Chinese population.

The mechanism underlying our findings are not known explicitly but could be related to the following factors. It has been suggested that insulin resistance alters glycolysis and renal handling of uric acid, thereby increasing the diversion of glycolysis inter-mediates to uric acid and decreasing urine uric acid clearance [33, 34]. The DASH diet has been proved to improve insulin resistance and increase insulin sensitivity significantly, and thus may have favourable effects on uric acid excretion [19, 35]. Substituting plant proteins of legumes and nuts for animal protein sources in the DASH diet may also be a reason for our results. High meat consumption, particularly red meat and seafood, have long been documented as a risk factor for HUA [10]. In contrast, higher soy food intake was found to be linked to lower serum uric acid levels in adults by a recent cross-sectional research conducted in Henan Province, China [36]. Previous studies have shown that although legumes are considered a purine-rich food, some special constituents such as phytic acid and polyphenols may have a beneficial influence on uric acid levels [37, 38]. In addition, actual purine intake may be lower due to the purines loss of soy foods during processing and cooking [39, 40]. At present, studies on the relation between nuts consumption and HUA risk are limited, but peanuts, one of the nuts commonly found in China, has been documented to have an positive impact on SUA levels [10]. Whole grain, as a important component of the DASH diet, may also contribute to the decrease in SUA levels. The consumption of whole grains improves glucose tolerance and peripheral insulin sensitivity, which may lead to a decrease in SUA levels [41]. Cereal fiber rich in whole grains has also been found to be negatively linked with blood uric acid contents and the odds of HUA in previous national representative studies in China and the United States [42, 43].

In subgroup analysis, we established that the effect of DASH diet were more strongly associated with HUA among men than women. This may be because men generally have higher levels of SUA than women, while the DASH diet may have a greater effect on those with higher levels of SUA [20]. Our study also explored the differences in the association between DASH diet and HUA among residents of different ethnic groups in China, and the results showed that the association was stronger among non-Han Chinese. Genetic factors may play an indispensable role in the risk association [10]. In addition, we established that the effect of DASH diet on HUA was significantly stronger in rural residents relative to urban residents. Higher levels of residential greenness and better sleeping patterns were documented to be related with lower risk of HUA in previous Chinese studies [44–46]. Therefore, we speculate that differences in living environment and lifestyle between urban and rural areas may be attributable to the effect modification.

The DASH diet is based on the Western dietary pattern and was initially developed for the American population. Over the past three decades, the dietary patterns of Chinese adults have shown a trend toward Western, mainly in the form of reduced intakes of vegetables and whole grains and increased intakes of red meat, processed meat, sugary drinks, saturated fats, and alcohol [47]. In this situation, the DASH dietary pattern as a healthy nutritional strategy may also contribute to the quality of the diet of the Chinese population. However, given the cultural and dietary differences, we still need to develop a modified DASH diet more suitable for our population, as Japan and Korea have done [17]. For example, since the Chinese diet has a low dairy product intake, we can increase calcium intake by increasing traditional foods such as beans and tofu. The development of the DASH diet for the Chinese population considering traditional staples, dishes, and other dietary habits will focus on our subsequent studies.

There are several strengths to our study. First, this is the first research premise to assess the relationship of the DASH diet with SUA levels and the odds of HUA among Chinese adults using a nationally representative sample. Thus, our results can be generalizable to the whole Chinese adults population. Second, the inverse association of the DASH diet with SUA contents and HUA was sustained following adjustment for numerous prospective confounding variables. Thirdly, Standardized methods as well as data collecting processes, and also training along with stringent quality control for all participants, assured the credibility of this research work’s findings.

Several limitations are also present in this research premise. Due to the cross-sectional aspect of this premise, we were unable to confirm a causal relationship of the DASH diet with SUA levels and HUA. Those conclusions would need to be confirmed by longitudinal studies. In addition, we could not exclude patients with gout who were finally enrolled in our study due to lack of information. According to the estimated prevalence of gout among Chinese adults in 2017 [48], there might be nearly 285 potential (0.429%) gout patients in our research. Therefore, the use of urate-lowering therapies and diuretics of these patients may still have an impact on our results although the number of these patients was small. At last, since we estimated levels of dietary intake using the 24-hour dietary recall method, long-term dietary intake may not accurately be reflected in our findings. Nevertheless, relative to the questionnaire of food frequency, the 24-hour recall yielded greater data on the kind and amount of food consumed, potentially reducing the possibility of under- or over-estimating nutrient intake levels [49].

## Conclusions

Herein, a negative relationship of DASH diet with SUA contents and the odds of HUA among the Chinese adult population was reported. This association was stronger among man, non-Han Chinese and rural residents. These findings may provide new dietary strategies into the prevention and management of HUA.

## Supplementary Information

**Additional file 1: Table S1.** Nutrient targets for DASH score.

## Data Availability

The data are not allowed to be disclosed according to the National Institute for Nutrition and Health, Chinese Center for Disease Control and Prevention.

## Abbreviations

SUA: Serum uric acid
HUA: Hyperuricemia
DASH: Dietary Approaches to Stop Hypertension
BMI: Body mass index
NCDs: Noncommunicable diseases
