# Supplementary material for: Association between dietary approaches to stop hypertension (DASH) diet and hyperuricemia among Chinese adults: findings from a nationwide representative study
Source: Nutr J. 2023 Mar 29;22:21. doi: 10.1186/s12937-023-00845-w (PMC10053091; doi:10.1186/s12937-023-00845-w)
Supplement: Supplementary file 1 — Additional file 1: Table S1. Nutrient targets for DASH score. [file 12937_2023_845_MOESM1_ESM.docx]

**Table S1.** Nutrient targets for DASH score

| Nutrients | DASH  Score Target | Intermediate  Target |
| --- | --- | --- |
| Saturated fat (% of energy) | 6 | 11 |
| Total fat (% of energy) | 27 | 32 |
| Protein (% of energy) | 18 | 16.5 |
| Cholesterol (g/1000 kcal) | 71.4 | 107.1 |
| Fiber (g/1000 kcal) | 14.8 | 9.5 |
| Magnesium (g/1000 kcal) | 238 | 158 |
| Calcium (g/1000 kcal) | 590 | 402 |
| Potassium (g/1000 kcal) | 2238 | 1534 |
| Sodium (g/1000 kcal) | 1143 | 1286 |
